# Supplementary material for: Untangling the effects of multiple exposures with a common reference group in an epidemiologic study: A practical revisit
Source: PLoS One. 2023 Dec 15;18(12):e0295915. doi: 10.1371/journal.pone.0295915 (PMC10723729; doi:10.1371/journal.pone.0295915)
Supplement: Attachment — Submitted filename: Response to reviewers.docx [file pone.0295915.s002.docx]

PONE-D-23-13611: Untangling the Effects of Multiple Exposures with a Common Reference Group in an Epidemiologic Study: A Practical Revisit

*PLOS ONE*

**Author Responses to Comments from Reviewers and Editor**

| **Comments** | **Response** |
| --- | --- |
| **Academic Editor’s Comments and Author Responses** | |
| Please work on your introduction, purpose of study and background by adding more precise literature. | Thank you for your suggestion. We have moved a paragraph reviewing the current literature on this topic from the Discussion section to the Introduction section.  We have also revised and expanded the Introduction section to explain the purpose of our work more clearly. |
| If possible use an editor to edit your work before resubmitting to avoid typo errors.  Also see below comments from a reviewer and address them. | All three co-authors have copy-edited the manuscript to ensure it is free of typo errors.  We have responded to comments from Reviewer #1, shown below. |
| Please include the following items when submitting your revised manuscript:   - A rebuttal letter that responds to each point raised by the academic editor and reviewer(s). You should upload this letter as a separate file labeled 'Response to Reviewers'. - A marked-up copy of your manuscript that highlights changes made to the original version. You should upload this as a separate file labeled 'Revised Manuscript with Track Changes'. - An unmarked version of your revised paper without tracked changes. You should upload this as a separate file labeled 'Manuscript'. | - This document serves as the letter responding to each point raised by the academic editor and reviewer. - A marked-up copy showing detailed revisions to the manuscript has been included. - An unmarked version without track changes have also been included. |
| If you would like to make changes to your financial disclosure, please include your updated statement in your cover letter. Guidelines for resubmitting your figure files are available below the reviewer comments at the end of this letter. | There are no changes to the financial disclosure. |
| If applicable, we recommend that you deposit your laboratory protocols in protocols.io to enhance the reproducibility of your results. Protocols.io assigns your protocol its own identifier (DOI) so that it can be cited independently in the future. For instructions see: <https://journals.plos.org/plosone/s/submission-guidelines#loc-laboratory-protocols>. Additionally, PLOS ONE offers an option for publishing peer-reviewed Lab Protocol articles, which describe protocols hosted on protocols.io. Read more information on sharing protocols at <https://plos.org/protocols?utm_medium=editorial-email&utm_source=authorletters&utm_campaign=protocols>. | There are no laboratory protocols in this manuscript. |
| When submitting your revision, we need you to address these additional requirements.  1. Please ensure that your manuscript meets PLOS ONE's style requirements, including those for file naming. The PLOS ONE style templates can be found at  <https://journals.plos.org/plosone/s/file?id=wjVg/PLOSOne_formatting_sample_main_body.pdf> and  <https://journals.plos.org/plosone/s/file?id=ba62/PLOSOne_formatting_sample_title_authors_affiliations.pdf> | We have reviewed PLOS ONE’s style requirements, and revised the manuscript accordingly to conform to the styles. |
| 2. In your Data Availability statement, you have not specified where the minimal data set underlying the results described in your manuscript can be found. PLOS defines a study's minimal data set as the underlying data used to reach the conclusions drawn in the manuscript and any additional data required to replicate the reported study findings in their entirety. All PLOS journals require that the minimal data set be made fully available. For more information about our data policy, please see <http://journals.plos.org/plosone/s/data-availability>.  Upon re-submitting your revised manuscript, please upload your study’s minimal underlying data set as either Supporting Information files or to a stable, public repository and include the relevant URLs, DOIs, or accession numbers within your revised cover letter. For a list of acceptable repositories, please see <http://journals.plos.org/plosone/s/data-availability#loc-recommended-repositories>. Any potentially identifying patient information must be fully anonymized.  Important: If there are ethical or legal restrictions to sharing your data publicly, please explain these restrictions in detail. Please see our guidelines for more information on what we consider unacceptable restrictions to publicly sharing data: <http://journals.plos.org/plosone/s/data-availability#loc-unacceptable-data-access-restrictions>. Note that it is not acceptable for the authors to be the sole named individuals responsible for ensuring data access.  We will update your Data Availability statement to reflect the information you provide in your cover letter. | We have made the data sets underlying this work available (**S1 File**), and have acknowledged the sources of the data sets. |
| 3. Please review your reference list to ensure that it is complete and correct. If you have cited papers that have been retracted, please include the rationale for doing so in the manuscript text, or remove these references and replace them with relevant current references. Any changes to the reference list should be mentioned in the rebuttal letter that accompanies your revised manuscript. If you need to cite a retracted article, indicate the article’s retracted status in the References list and also include a citation and full reference for the retraction notice. | We have reviewed the references to ensure they are complete and correct. To our knowledge, none of our cited references have been retracted. |
| **Reviewer #1 Comments and Author Responses** | |
| The authors describe method of analyzing epidemiological data using a common reference group, this was to avoid misinterpretation of data and consequential shifting reference problem that would follow. The article is well written, easy to follow, and the language is clear even for unseasoned researcher to understand. | Thank you for your observation. We appreciate your kind review and suggestions. |
| Though, felt the introduction lacked details about the purpose and background of the review. I would recommend that the authors add more information and literature reviews to expand how the review would improve the use of common reference group in analysis of epidemiologic studies. | Thank you for your suggestion. We have moved a paragraph reviewing the current literature on this topic from the Discussion section to the Introduction section.  We have also revised and expanded the Introduction section to explain the purpose of our work more clearly. |
| Overall, the paper is sound and would make an interesting addition to literature to augment epidemiological research. | Again, many thanks for your suggestions. |
